# The Hour at Which Death Most Usually Occurs

**Published:** 1890-01

**Authors:** 


### Editorial, Etc.

---

THE HOUR AT WHICH DEATH MOST USUALLY OCCURS —INTERESTING FIGURES.—John Francis Burns, M. D., writes as follows: A very general opinion is entertained, says the New York *Medical Journal*, by medical practitioners and others engaged in caring for the sick that the greatest number of deaths occurring in individuals afflicted with disease takes place during the hours immediately succeeding midnight and preceding the dawn. This opinion most probably originates in part from imperfect observation, and partly from a misapplication of the physiological law governing the lowest period of vitality in the healthy individual. The rule is said to be particularly true in those suffering from chronic exhausting diseases, and deductions have been drawn from these impressions which have served to regulate the administration of stimulants in such cases, it being said, "if six ounces of whiskey be needed in twenty-four hours, four should be administered from 2 to 6 A. M., for then is vitality in the human being at its lowest," and "more deaths occur then than at any other period."

Such expressions may be found scattered through works on materia medica and therapeutics, and in many of the text books on the practice of medicine. The idea finds expression also in the lectures of teachers in our colleges, and usually leaves a well-grounded impression on the mind of the medical student, which is apt to remain a permanent one. I accepted this teaching at college because I had neither the means nor the time to verify or disprove it to my own satisfaction. Yet I always doubted the correctness of the conclusions drawn, and, to settle the doubt in my mind, since entering on my duties at the hospital I have collected the statistics given below, which I find do not agree with this generally accepted idea. Thinking that they may interest others, and that the small mistakes in a subject are sometimes and to certain people as important as the greater, I present them.

The statistics are taken from the records of the Charity Hospital on the one hand, and from the New York board of health on the other. The former are mainly of deaths occurring in those afflicted with chronic exhausting diseases; the latter, in those dying from the acute exanthemate. The former represents all the deaths at the hospital for a period of nearly ten years, irrespective of sex, age, disease, or condition; the latter, all the deaths occuring in the city and county of New York from the acute contagious diseases. At the hospital the records of death are kept with great care, and I am sure can be taken as a fair test. I have no doubt that the health authorities' records are also accurate, but they are the result of individual reporters, so that they are not so reliable as those of the hospitals. There are many circumstances that should greatly tend to increase the death rate at night in a large public hospital, principal among which is the great vitiation of the atmosphere during this period. During the night all the patients are confined to the ward, and ventilation is apt to be neglected. This must certainly have a very depressing effect on those suffering with pulmonary affections, and on those in whom disease has effected extensive alterations in the physical and chemical characters of the blood. This alone should greatly tend to increase the number of deaths at night, and, if there was any truth in the accepted notion the records should show quite a preponderance of deaths happening at night. The contrary is, however, the rule, the figures showing 27 cases fewer from 6 P. M. to 6 A. M. than for the corresponding twelve hours of the day.

Again, from 2 to 6 P. M. there were 66 more deaths than from 2 to 6 A. M. The total number of deaths in the list of acute diseases for the twelve hours from 6 P. M. to 6 A. M. is 169 less than for the corresponding period during the day. The hours from 2 to 6 A. M. in this list shows 53 cases more than for the corresponding period in the afternoon; this in nearly 4,000 cases is very slight. In the chronic cases the greatest number of deaths at any one hour was at 4 P. M., with 2 and 5 P. M. and 6 A. M. close following; the greatest in the acute list at 3 A. M., with 11 A. M. and P. M. close following. The lowest number in the acute list at 12 M., (midnight,) that hour so dreaded in the sick room by attendants, and to which a good deal of superstition attaches. It is noticeable that the number for this hour is exceedingly low—about half of the average number. In the chronic diseases the lowest number appears at 9 A. M. In the Chronic cases the number dying from 9 A. M. to 12 M. (noon) seems relatively low compared with the same period in the acute list. I have used all the figures available at the hospital, and I only stopped when the death books available were exhausted. I only sought the health board's statistics for the purpose of comparison, and as the figures run up quickly, I thought two records would serve as well as a longer period. In making the collections I noticed that the figures did not vary essentially throughout. There was always a preponderance of deaths in favor of the hours of the day, while the individual hours would vary by comparison at different periods.

Deaths occuring at Charity Hospital, Blackwell's Island, during the past ten years, by hours:

| 6 A. M. to 6 M. M. | | 6 P. M. to 6 A. M. | |
|---|---|---|---|
| 6 A. M. | 205 | 6 P. M. | 187 |
| 7 A. M. | 178 | 7 P. M. | 187 |
| 8 A. M. | 180 | 8 P. M. | 181 |
| 9 A. M. | 138 | 9 P. M. | 197 |
| 10 A. M. | 165 | 10 P. M. | 165 |
| 11 A. M. | 169 | 11 P. M. | 172 |
| 12 noon | 175 | 12 midnight | 165 |
| 1 P. M. | 178 | 1 A. M. | 173 |
| 2 P. M. | 204 | 2 A. M. | 175 |
| 3 P. M. | 191 | 3 A. M. | 181 |
| 4 P. M. | 209 | 4 A. M. | 194 |
| 5 P. M. | 206 | 5 A. M. | 194 |
| Total | 2.198 | Total | 2.171 |

Difference in favor of the hours of the day, 27.

Deaths occurring in the foregoing table from 2 to 6 A. M. and 2 to 6 P. M.

| 2 to 6 A. M. | | 2 to 6 P. M. | |
|---|---|---|---|
| 2 A. M. | 175 | 2 P. M. | 204 |
| 3 A. M. | 181 | 3 P. M. | 191 |
| 4 A. M. | 194 | 4 P. M. | 209 |
| 5 A. M. | 194 | 5 P. M. | 206 |
| Total | 744 | Total | 810 |

Difference in favor of afternoon hours, 66.

Deaths from acute contagious diseases for two years reported to the New York board of health by hours.

| 6 A. M. to 6 P. M. | | 6 P. M. to 6 A. M. | |
|---|---|---|---|
| 6 A. M. | 464 | 6 P. M. | 474 |
| 7 A. M. | 386 | 7 P. M. | 430 |
| 8 A. M. | 420 | 8 P. M. | 369 |
| 9 A. M. | 434 | 9 P. M. | 411 |
| 10 A. M. | 450 | 10 P. M. | 420 |
| 11 A. M. | 512 | 11 P. M. | 502 |
| 12 noon | 448 | 12 midnight | 243 |
| 1 P. M. | 436 | 1 A. M. | 479 |
| 2 P. M. | 428 | 2 A. M. | 434 |
| 3 P. M. | 436 | 3 A. M. | 506 |
| 4 P. M. | 493 | 4 A. M. | 499 |
| 5 P. M. | 482 | 5 A. M. | 453 |
| Total | 5.389 | Total | 5.220 |

Difference in favor of the hours of the day, 169.

Deaths occuring in the foregoing list from 2 to 6 A. M. and 2 to 6 P. M.

| 2 to 6 A. M. | | 2 to 6 P. M. | |
|---|---|---|---|
| 2 A. M. | 434 | 2 P. M. | 428 |
| 3 A. M. | 506 | 3 P. M. | 436 |
| 4 A. M. | 499 | 4 P. M. | 493 |
| 5 A. M. | 453 | 5 P. M. | 482 |
| Total | 1.892 | Total | 1.839 |

Difference in favor of early morning hours, 53.

From these 15.000 cases, extending over a period of twelve years, it would appear that death occurs seemingly without any particular predilection for any certain hour, and that the number of deaths for each hour is very evenly proportioned, considering the large number of cases taken and the time covered. The only very positive conclusions I have formed from the figures are: 1. That the idea that more deaths take place in the early morning hours is an erroneous one. 2. If stimulants are to be pushed into disease during these hours, the practice must be justified upon some other ground than to avert the possibility of danger supposed to be very probable at this period. 3. That the vitality of an individual in disease is not regulated by the same influences or subject to the same laws that govern the vitality of a healthy human being, the normal equilibrium maintained in health between the mental and physical states being altered.

---

